# Epigallocatechin gallate (EGCG) modulates senescent endothelial cell-monocyte communication in age-related vascular inflammation

**DOI:** 10.3389/fcvm.2024.1506360

**Published:** 2025-01-21

**Authors:** Sarvatit Patel, Kai Ellis, Corey A. Scipione, Jason E. Fish, Kathryn L. Howe

**Affiliations:** ^1^ Toronto General Hospital Research Institute, University Health Network, Toronto, ON, Canada; ^2^ Department of Molecular Genetics, University of Toronto, Toronto, ON, Canada; ^3^ Department of Genetics and Genome Biology, SickKids Research Institute, Toronto, ON, Canada; ^4^ Institute of Medical Science, University of Toronto, Toronto, ON, Canada; ^5^ Department of Laboratory Medicine and Pathobiology, University of Toronto, Toronto, ON, Canada; ^6^ Peter Munk Cardiac Centre, University Health Network, Toronto, ON, Canada; ^7^ Division of Vascular Surgery, Department of Surgery, University of Toronto, Toronto, ON, Canada

**Keywords:** aging, cardiovascular disease, endothelial senescence, endothelial-monocyte communication, extracellular vesicles, inflammation, epigallocatechin gallate (EGCG)

## Abstract

Aging significantly affects intercellular communication between vascular endothelial cells (ECs) and hematopoietic cells, leading to vascular inflammation and age-associated diseases. This study determined how senescent ECs communicate with monocytes, whether extracellular vesicles (EVs) released from senescent ECs affect monocyte functions, and investigated the potential for epigallocatechin-3-gallate (EGCG), a flavonoid in green tea, to reverse these effects. Human umbilical vein endothelial cells (HUVECs) were treated with Etoposide (10 µM, 24 h) to induce senescence, followed by EGCG (100 µM, 24 h) treatment to evaluate its potential as a senotherapeutic agent. The interaction between ECs and monocytes was analyzed using a co-culture system and direct treatment of monocytes with EC-derived EVs. EGCG reduced senescence-associated phenotypes in ECs, as evidenced by decreased senescence-associated (SA)-β-Gal activity and reversal of Etoposide-induced senescence markers. Monocytes co-cultured with EGCG-treated senescent ECs showed decreased pro-inflammatory responses compared to those co-cultured with untreated senescent ECs. Additionally, senescent ECs produced more EVs than non-senescent ECs. EVs from senescent ECs enhanced lipopolysaccharide (LPS)-induced pro-inflammatory activation of monocytes, whereas EVs from EGCG-treated senescent ECs mitigated this activation, maintaining monocyte activation at normal levels. Our findings reveal that EGCG confers anti-senescent effects via modulation of the senescent EC secretome (including EVs) with the capacity to modify monocyte activation. These findings suggest that EGCG could act as a senotherapeutic agent to reduce vascular inflammation related to aging.

## Graphical Abstract

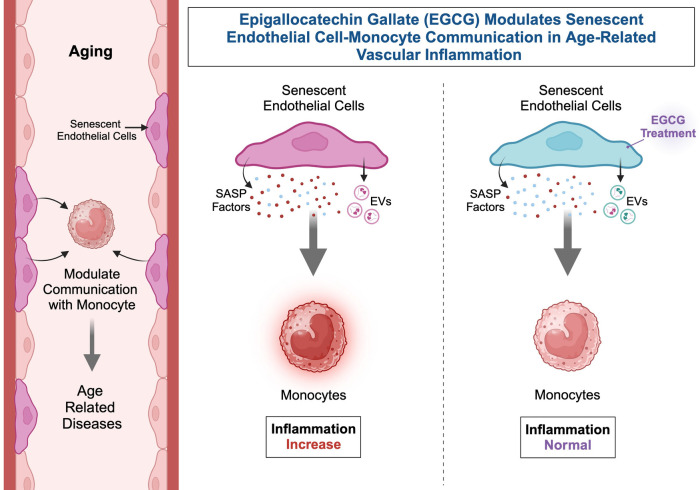

## 1 Introduction

Aging is primarily attributed to the progressive accumulation of damage in cells, organelles, and macromolecules. The hemothelium is comprised of the hematopoietic system (blood cells) and the vascular endothelium (a monolayer of cells lining all blood vessels), which maintain continuous interactions throughout an individual's lifespan (1). In numerous conditions, including cardiovascular diseases (CVDs), the hemothelium is dysregulated (1–3). Several age-related CVDs have been associated with endothelial dysfunction resulting from endothelial senescence (4). Endothelial senescence is marked by an age-related decline in endothelial function, which encompasses impaired regulation of vasodilation, blood coagulation, oxidative stress, inflammation, immune cell infiltration, as well as glucose and lipid metabolism (4). Age-related inflammation, often termed “inflammaging”, involves the contribution of monocytes and macrophages to inflammatory processes, significantly contributing to the compromised immune function observed with advancing age (5). Given an aging population and the rising incidence of CVD, it is imperative to elucidate how aging contributes to alterations within the hemothelium.

The interactions between monocytes and endothelial cells (ECs) regulate vascular and tissue remodelling, contributing to CVDs (6). Monocytes interact with ECs to regulate processes such as inflammation, angiogenesis, and tissue remodelling (7). Monocytes undergo classical activation in response to pro-inflammatory stimuli such as interferon-γ or bacterial lipopolysaccharide (LPS), leading to the release of pro-inflammatory cytokines and reactive oxygen/nitrogen species, promoting an M1-like pro-inflammatory response (8–11). Age-related changes in monocytes include distinct inflammatory gene expression profiles and increased production of pro-inflammatory cytokines [e.g., interleukin (IL)-8, IL-12p70] in response to Toll-like receptors (TLR) 4 and TLR2/1 stimulation (12). So far, the communication of senescent ECs with circulating monocytes during aging is largely unknown.

Cellular senescence is associated with the development of a multicomponent senescence-associated secretory phenotype (SASP) (13–16). Canonical SASP factors comprise a collection of cytokines, chemokines, growth factors, and proteases released by senescent cells, which initiate inflammation, wound healing, and growth responses in nearby cells (17–19). Evidence from ECs and other cell types suggests that secreted SASP factors can induce senescence in neighbouring cells (20, 21). As ECs lose their proliferative potential and become senescent, they activate the SASP, which includes inflammatory pathways such as nuclear factor kappa B (NF-κB) and the secretion of inflammatory cytokines and reactive oxygen species (22–24). Senescent ECs and their secreted factors significantly contribute to arterial dysfunction and the pathophysiology of various cardiometabolic diseases (25). This indicates that the secretome from senescent ECs has the potential for altered communication with blood monocytes, and may contribute to age-related diseases. ECs can communicate by releasing extracellular vesicles (EVs), which are nanoparticles representing a novel paradigm in cell-cell communication. These lipid bilayer-encapsulated vesicles carry diverse cargo, including lipids, proteins, and transcripts, which can influence cellular functions and signal disease states (26). EVs are released into body fluids by most cell types, including senescent ECs and they contribute to processes such as vascular calcification, inflammation, cellular senescence, endothelial dysfunction, and fibrosis (27). EV cargo can contain SASP proteins, which can be transported to target cells, altering their phenotypes. Previous studies, including our own, have demonstrated that cross-talk between ECs and monocytes/macrophages occurs partly through the secretion of EVs (8, 28–31). It is known that EVs released from IL-1β stimulated ECs can communicate with monocytes and encourage inflammatory phenotypes (8, 32). While EVs released from IL-1β stimulated ECs (28) or during sepsis (33) can reprogram monocytes, it is unknown whether EVs released from senescent ECs similarly communicate with monocytes and importantly, whether this can be reversed. We hypothesize that the secretome, specifically EVs, released from senescent ECs may enhance monocyte-driven inflammation and that this can be reversed with senotherapeutics.

Epigallocatechin gallate (EGCG), the primary catechin in green tea, is associated with numerous health benefits, including impacts on cell senescence, aging, and age-related diseases (34–37). Preclinical studies show that EGCG exerts senolytic and senomorphic effects (38, 39). Research on murine models of aging has identified plasma EV-miRNAs associated with aging, which appear to be reversed by senolytics (40). However, whether these effects are derived explicitly from ECs remains unclear. Additional studies have demonstrated that EGCG consumption can produce anti-cellular senescence and anti-skin aging effects in mouse tissues (35, 41). This study employed an *in vitro* model of senescent ECs to assess the therapeutic potential of EGCG in reversing endothelial senescence and its impact on EC-monocyte communication. We utilized a co-culture system to assess the role of the secretome from senescent ECs, including the specific contribution of purified senescent EC-derived EV treatments, to comprehensively evaluate how senescence-driven endothelial changes influence monocyte dynamics. The reversibility of these effects was subsequently assessed by EGCG treatment, providing novel insights into EV mediated cellular interactions in the context of aging and inflammation.

## 2 Methods

### 2.1 Primary cell culture

Human umbilical vein endothelial cells (HUVECs; ECs, LONZA) were cultured in Endothelial Cell Medium (ECM, ScienCell) supplemented with 5% Fetal Bovine Serum (FBS) and Endothelial Cell Growth Supplement (ScienCell). ECs were detached using Trypsin-EDTA (0.25%) and used at passages 5–7. THP1 monocytes were cultured in RPMI 1640 medium (WISENT) with 10% FBS (WISENT). For the EC senescence model, ECs (seeded in 6-well plates at a density of 80,000 cells/well) were treated with Dimethylsulfoxide (DMSO) as a control or Etoposide (10 µM, Sigma-Aldrich) for 24 h to induce senescence, as this is a dose and time that has been used in other cell models (42, 43). Subsequently, the senescent ECs were treated with EGCG [100 µM, Sigma-Aldrich (38)], for 24 h, based upon a treatment time used in other EC models (44). RNA and culture supernatant media were collected from the ECs for qRT-PCR and multiplex cytokine analysis, respectively.

### 2.2 β-Galactosidase assay

As described above, ECs were plated onto 6-well plates (80,000 cells/well). Cellular senescence was identified using β-galactosidase activity detected by histochemical staining, performed as per the instructions of the Senescence-Associated β-Galactosidase Staining Kit (Cell Signaling). Images of stained cells were captured using a Nikon ECLIPSE Ti microscope. For each experimental group, three biological replicates (each with two technical replicates) were analyzed. Four images were captured per replicate. The percentage of SA-β-galactosidase positive cells was quantified using Image J software. Data from eight images per biological replicate were combined to calculate the average percentage of SA-β-galactosidase-positive cells. The results represent the average stained area per cell across three biological replicates.

### 2.3 Co-culture experiments

For co-culture experiments, ECs were seeded in 10 cm plates ± DMSO, Etoposide, or Etoposide + EGCG. Cells were collected, washed and counted before co-culture experiments. ECs were then plated onto 6-well plates (500,000 cells/well) and cultured in complete ECM for 2 h to allow for adherence. After 2 h, THP1 cells (400,000 cells/well) were seeded in the upper chamber of transwell membrane inserts (1 µm pore size selected to ensure passage of whole secretome, STERLITECH), and co-cultured with control ECs as previously published (8), or senescent ECs (senECs) or EGCG-treated senECs in complete ECM. THP1 cells were grown in complete ECM without ECs (mono-culture) as a control. Following 24 h of mono-culture or co-culture, THP1 cells were removed and stimulated with 100 ng/ml LPS (Sigma-Aldrich) for 2 h, as before (8).

### 2.4 Multiplex analysis of cytokines

Culture media (2 ml) was collected following treatments, and 150 µl was used to measure concentrations of cytokines, chemokines, and growth factors using the Human Cytokine Panel A 48-Plex Discovery Assay® from Eve Technologies (Calgary, AB, Canada). Multiplex quantification was performed with the Luminex™ 200 system (Luminex, Austin, TX, USA).

### 2.5 RNA isolation, quality control, and sequencing

RNA was isolated using TRIzol™ Reagent from THP1 cells (mono-culture and co-culture) after LPS stimulation according to the manufacturer's instructions. RNA concentration and quality was measured using the Agilent 2100 Bioanalyzer (Total RNA Pico Chip). Preparation of RNA library and mRNA sequencing was completed by Novogene Co. LTD (Sacramento, CA, USA) using the Illumina NovaSeq X Plus Series (PE150) Sequencing System of samples meeting minimum input requirements [amount ≥ 200 ng, RIN > 8, purity (A260/280 ≥ 1.8, A260/230 ≥ 1.8)]. Paired-end sequencing (150 bp) with a minimum read depth of 30 million read pairs per sample was targeted. Differentially expressed mRNA transcripts analysis were completed by Novogene Co. LTD. Briefly, clean reads were obtained by removing reads containing adapters, greater than 10% unidentifiable bases, and low-quality reads (Qscore of over 50% bases ≤ 5). Clean reads were mapped to a reference genome using HISAT2. Starting from Novogene's count matrix, detectable genes were filtered based on expression values (sum of counts ≥ 10), and RNA differential expression was performed using the DEseq2 package (version 1.44) for R (version 4.4.1). Significance was called as *p*adj < 0.05, with no fold change cutoff. FPKM (Fragments Per Kilobase of transcript per Million mapped reads) read count normalization was performed using DESeq2. *Z*-score heatmaps were generated using the FPKM values of the selected genes. Gene set enrichment analysis (GSEA) was performed against the msigDB human hallmark genesets using the fgsea package (v1.3.0) for R, with ranking based on the wald statistic.

### 2.6 Real-time quantitative reverse-transcriptase polymerase chain reaction (qRT-PCR)

RNA was isolated using TRIzol™ Reagent (Invitrogen) and reverse transcribed using the High-Capacity cDNA Reverse Transcription kit (Applied Biosystems). qRT-PCR was performed with SYBR green technology on a QuantStudio 5 Real-Time PCR system (Applied Biosystems) using LC 480 SYBR™ Green Master Mix (Applied Biosystems) as described previously (8, 28). Data were normalized to Glyceraldehyde 3-phosphate dehydrogenase (*GAPDH*) using the Delta-Delta Ct method. All qRT-PCR primers are listed in Supplementary Table 1.

### 2.7 Isolation and characterization of EVs

#### 2.7.1 EV collection

ECs (control, senECs, EGCG-treated senECs) were cultured on 15 cm plates and supplemented with ECM containing EV-depleted FBS [Ultrafiltration using spin columns (cytiva 100 kDa) at 3,000 g for 30 min, 0.22 µm filtered (45)] for 24 h. The culture medium was collected and pre-cleared by centrifugation at 500 g for 15 min and then at 3,000 g for 15 min. The supernatant was filtered with 0.22 µm filter (Millipore Sigma) and ultracentrifuged at 120,000 g for 180 min at 4°C, followed by washing of the EV pellet with PBS (0.22 µm filtered) at 120,000 g for 120 min at 4°C (Optima XP-90 Ultracentrifuge with Type 70.1 Ti Fixed-Angle Rotor, Beckman Coulter). The EV pellet was resuspended in PBS (0.22 µm filtered) and stored at −80°C.

#### 2.7.2 EV characterization

Nanoparticle Tracking Analysis (NTA) and Cryogenic Electron Microscopy (Cryo-EM) analyses were conducted following previously established protocols (28). Western blots were performed as described previously (28) using primary antibody against CD63 (1:750, sc-365604 Santa Cruz), CD81 (1:1000, 56039 Cell signaling), CD9 (1:200, sc-13118 Santa Cruz), Calnexin (1:200, sc-23954 Santa Cruz), or Alix (1:200, sc-53540 Santa Cruz).

### 2.8 EV treatment of THP1 cells

EVs from control ECs (EC-EVs), senECs (senEC-EVs), and EGCG-treated senECs (tsenEC-EVs) were isolated via ultracentrifugation from conditioned media. THP1 cells plated onto 96-well plates (300,000 cells in 250 µl media/well) were treated with PBS or EVs (EC-EVs, senEC-EVs, tsenEC-EVs) at 10^10^ EV particles/ml concentrations for 24 h, as before (28). After treatment, THP1 cells were stimulated with 100 ng/ml LPS for 2 h. RNA and culture supernatant media were collected from the THP1 cells for RT-PCR and multiplex cytokine analysis, respectively.

### 2.9 Statistical analyses

All statistical analyses were performed using GraphPad Prism 9 software. Data were analyzed using one- or two-way ANOVA, followed by the Tukey (or Bonferroni where indicated) multiple comparison test. Error bars represent the standard error of the mean (SEM). A *p*-value lower than 0.05 was considered statistically significant.

## 3 Results

### 3.1 EGCG alleviates senescence-associated phenotype in ECs

**Figure 1:**
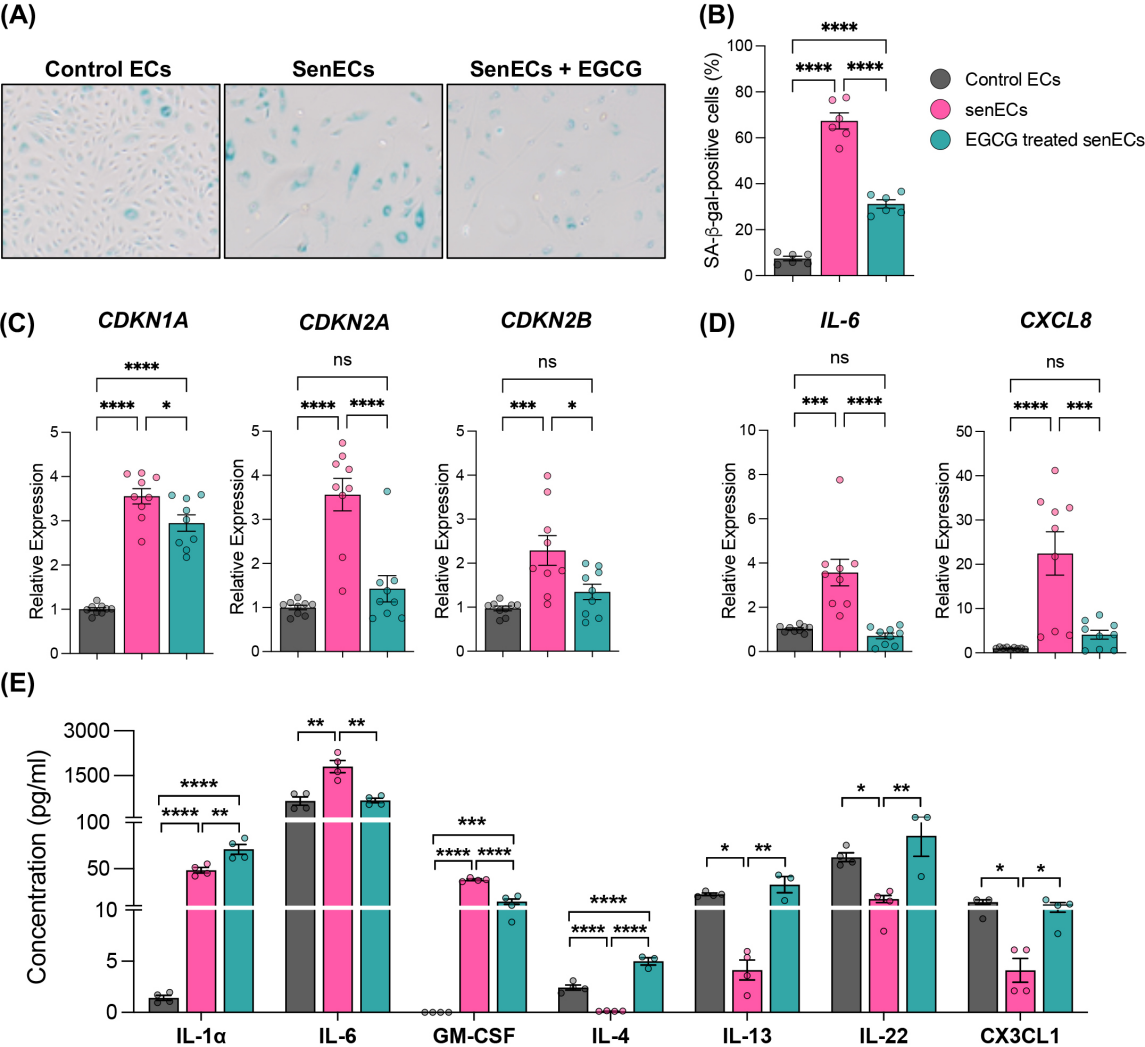
Antisenescence effects of EGCG on senescent ECs. **(A)** Representative cellular morphology and SA-β-gal staining of ECs under various conditions—(i) DMSO (control ECs), (ii) Treated with Etoposide (10 µM) for 24 h (senECs), and (iii) Treated with Etoposide (10 µM) for 24 h followed by EGCG (100 µM) treatment for 24 h (EGCG treated senECs). **(B)** Quantification of the percentage of SA-β-gal positive ECs. qRT-PCR analysis of **(C)** senescence-related genes *CDKN1A*, *CDKN2A*, *CDKN2B* and **(D)** senescence-associated secretory phenotype (SASP)-related genes *IL-6*, *CXCL8*. **(E)** Concentration of cytokines/chemokines (IL-1α, IL-6, GM-CSF, IL-4, IL-13, IL-22, and CX3CL1) in culture media from ECs across all experimental conditions was quantified using human cytokine and chemokine array. Data are given as ± SEM. *n* = 3–4 independent experiments. One-way ANOVA with a Tukey's multiple comparison test. ns, not significance; *p < 0.05, **p < 0.01, ***p < 0.001 and ****p < 0.0001.

One of the most well-characterized contributors to aging is senescent cells. A hallmark of senescent cells is the activity of senescence-associated β-galactosidase (SA-β-gal), as the presence of this active enzyme indicates the senescent state of cells (46, 47). Other well-established markers of cellular senescence are the elevated expression of cell cycle inhibitors p21 (*CDKN1A*), p16 (*CDKN2A*), and p15 (*CDKN2B*), along with SASP factors IL-6 and IL-8 (47). To test the anti-senescence properties of EGCG, an *in vitro* senescent cell model was established by treating ECs with Etoposide (10 µM) for 24 h. Treatment with Etoposide induced characteristic features of senescence, including increased SA-β-Gal activity (Figure 1A,B, Supplementary Figure 1A) and elevated levels of senescence- and SASP-related markers such as *CDKN1A*, *CDKN2A*, *CDKN2B*, *CXCL8*, and *IL6* (Figure 1C,D, Supplementary Figure 1B,C). This confirmed that Etoposide (10 µM, 24 h) treatment is a suitable senescence model. Subsequent treatment of senECs with EGCG (100 μM) for 24 h significantly reduced senescence-associated characteristics, including decreased SA-β-Gal activity (Figure 1A,B), compared to vehicle control. EGCG treatment also reversed the elevated expression of senescence-associated genes, including *CDKN1A*, *CDKN2A*, *CDKN2B*, *CXCL8*, and *IL6* (by 1.2-fold, 2.5-fold, 1.3-fold, 5.5-fold, 5-fold respectively), in senECs (Figure 1C,D). Additionally, EGCG supplementation was able to blunt the senescence induced upregulation of cell adhesion molecules *VCAM1* and *SELE*, but not *ICAM1*, which could suggest a reduction in monocyte adhesion (Supplementary Figure 2). Comparison of EGCG treatment to control ECs showed no difference in senescence-associated markers (Supplementary Figure 2).

SASP factors (pro-inflammatory cytokines, chemokines, and growth factors) undergo dynamic changes during cellular senescence (48). We investigated whether EGCG plays a role in the secretion of these conventional markers of senescent programming (Figure 1E). The abundance of key components of the secretome (cytokines and chemokines), including IL-1α, IL-6, granulocyte-macrophage colony-stimulating factor (GM-CSF), IL-4, IL-13, IL-22, and C-C motif chemokine ligand (CX3CL1) were measured in cell media by multiplex assay. SenECs showed increased IL-1α, IL-6 and GM-CSF abundance and decreased IL-4, IL-13, IL-22, and CX3CL1 compared to control ECs (Figure 1E). In contrast, treatment of senECs with EGCG led to a reduction in IL-6 (∼3-fold) and GM-CSF levels while increasing the expression of IL-4, IL-13, IL-22, and CX3CL1 in comparison to untreated senECs. These results suggest that EGCG treatment impairs the pro-inflammatory secretory phenotype and enhances the anti-inflammatory response in senECs. Overall, these data indicate that EGCG treatment mitigates the SASP and highlights its potential immunomodulatory effects on senescent ECs.

### 3.2 EGCG decreases monocytic proinflammatory responses induced by senescent ECs

**Figure 2:**
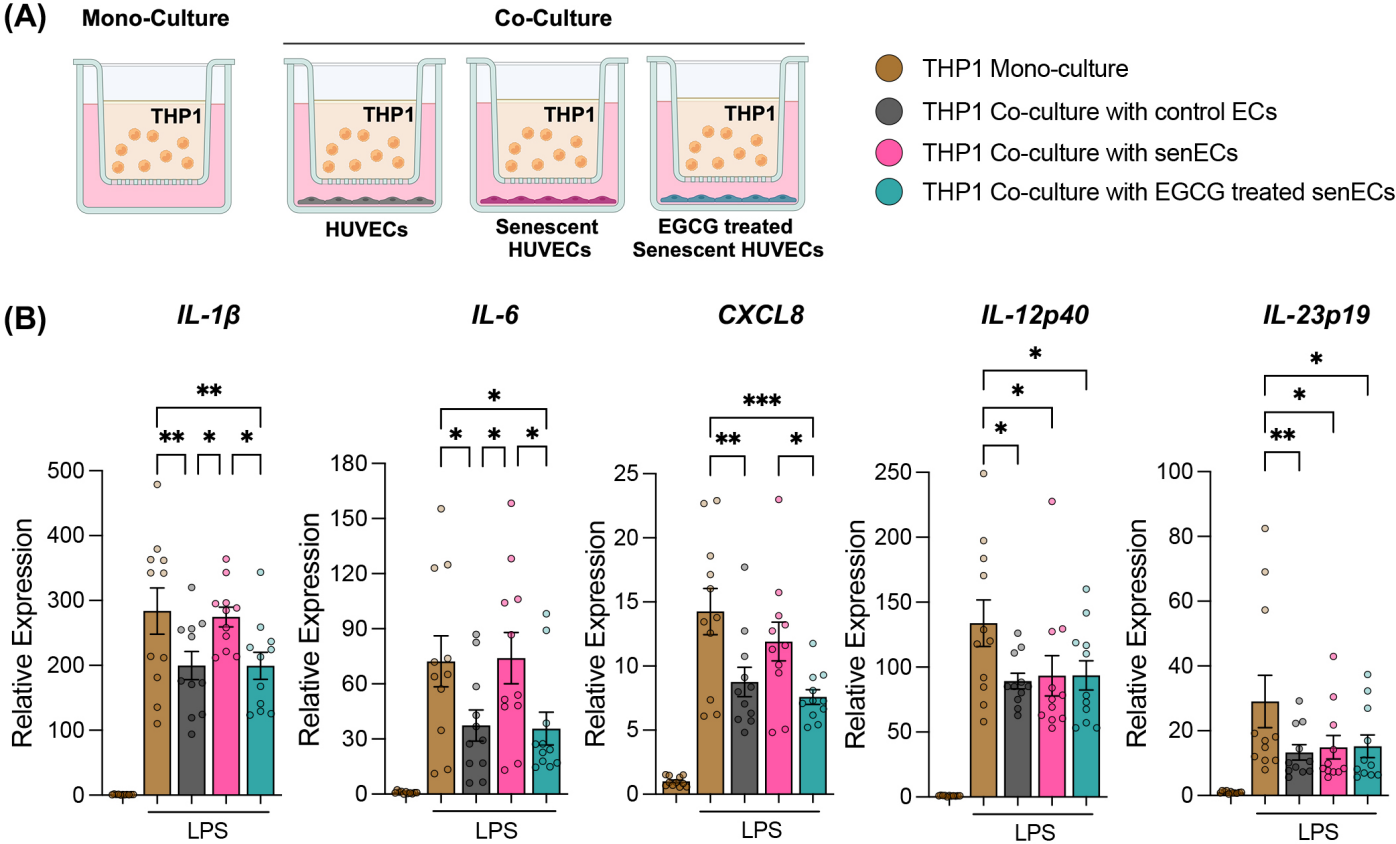
Co-culture with EGCG-treated senescent ECs suppresses monocyte activation. **(A)** Schematic of co-culture of monocytes (THP1) with control ECs, senECs, and EGCG-treated senECs using a transwell system (1 μm pore size). **(B)** The impact of co-culturing THP1 cells with ECs for 24 h was assessed by measuring the transcriptional expression of proinflammatory genes by THP1 cells after 2 h of LPS stimulation using RT-PCR. Pro-inflammatory markers: *IL-1β*, *IL6*, *CXCL8*, *IL-12p40*, and *IL-23p19*. Data are normalized to the GAPDH reference gene. Results are reported as the fold change relative to THP1 mono-culture (without LPS treatment). Data are presented as mean ± SEM. *n* = 4 independent experiments. Statistical analysis was performed using two-way ANOVA with Tukey's multiple comparison test. *p < 0.05, **p < 0.01, and ***p < 0.001.

To assess intercellular signalling between ECs and monocytes, we established a co-culture model wherein control ECs, senECs or EGCG-treated senECs were co-cultured with the human monocytic cell line THP1, with a 1-μm transwell filter serving as a physical barrier (Figure 2A). Following mono-culture or co-culture (24 h), THP1 cells were removed from the transwell inserts and stimulated with LPS. As expected (8), co-culture with control ECs suppressed monocyte activation, as evidenced by reduced induction of *IL-1β*, *IL6*, *CXCL8*, *IL-12p40*, and *IL-23p19* compared to THP1 mono-culture (Figure 2B). Conversely, co-culture with senECs elevated the expression of *IL-1β* and *IL6* compared to co-culture with control ECs (Figure 2B). Notably, co-culture with EGCG-treated senECs decreased expression of *IL-1β*, *IL6*, and *CXCL8* compared to co-culture with untreated senECs and THP1 mono-culture (by 1.4-fold, 2-fold, 1.7-fold respectively) (Figure 2B). Collectively, these findings suggest that THP1 co-culture with control ECs suppresses monocyte activation, which is negated by senECs and restored by EGCG treatment.

### 3.3 EGCG treatment negates the pro-inflammatory effect of senescent ECs on monocyte gene regulatory pathways

**Figure 3:**
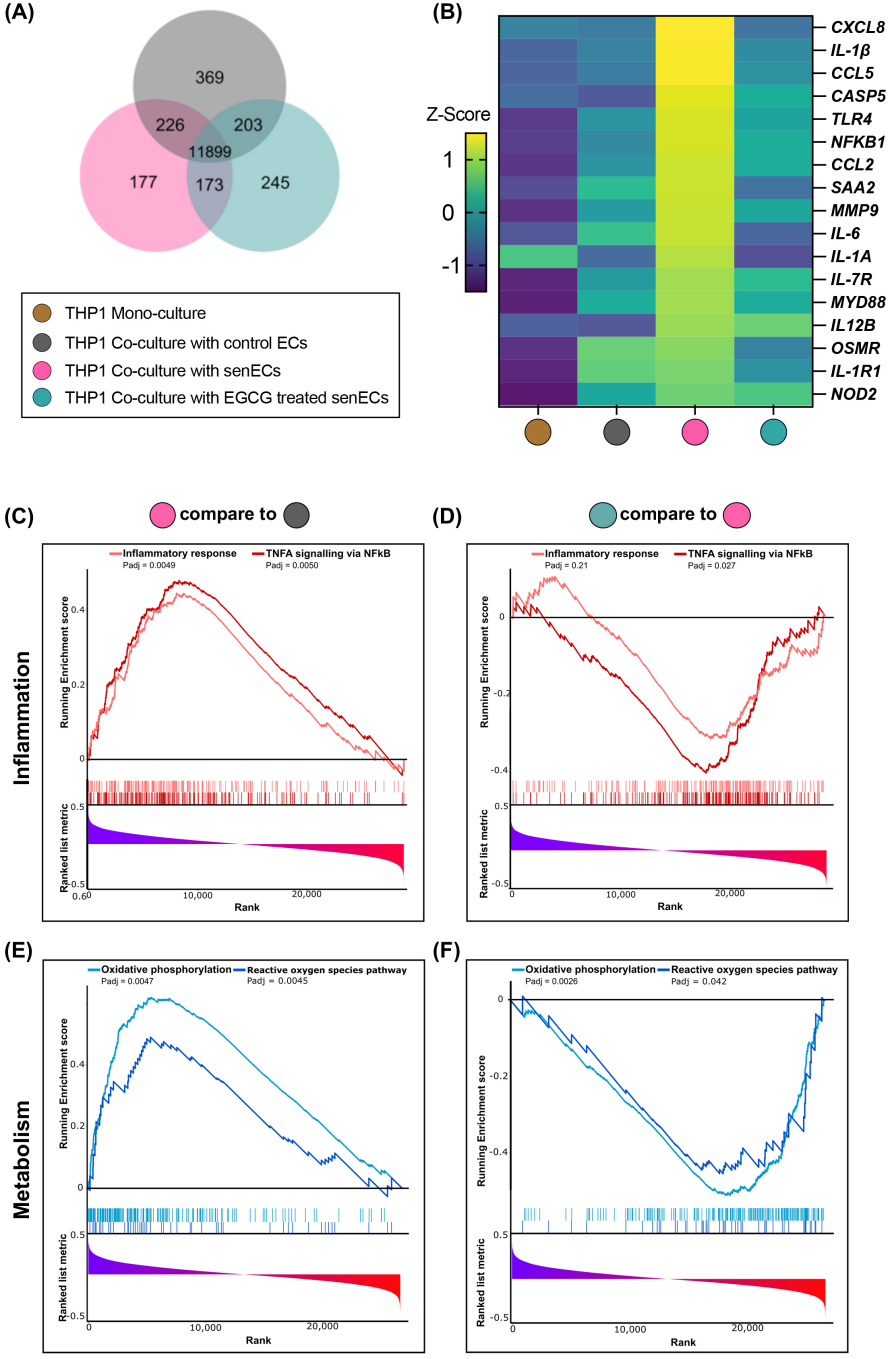
Effect of EGCG treatment on communication between senescent ECs and monocyte function. RNA sequencing was performed on samples collected after 24 h of co-culturing THP1 cells with control ECs, senECs, and EGCG-treated senECs. **(A)** Venn diagram depicting number of commonly and differentially expressed genes in comparisons of THP1 co-culture with control ECs, senECs, and EGCG-treated senECs. **(B)** Heatmap showing the expression profiles of a curated list of pro-inflammatory genes. Gene expression was assessed across different experimental conditions to evaluate changes in inflammatory responses. Expression values are represented as colours and range from yellow (high expression), green (moderate), to dark blue (lowest expression). Gene set enrichment analysis (GSEA) enrichment plots of inflammation-related gene sets comparing **(C)** THP1 cells co-cultured with senECs vs. control ECs, and **(D)** THP1 cells co-cultured with EGCG-treated senECs vs. untreated senECs. Metabolism-related gene sets are shown in **(E)** and **(F)** for the same comparisons indicated above.

To understand further how senescent ECs communicate with monocytes and affect cellular function, THP1 monocytes were co-cultured with control ECs, senECs, or EGCG-treated senECs for 24 h (Figure 2A). The cellular response of monocytes to co-culture with different ECs was delineated by RNA isolation from THP1 cells and RNA-sequencing. As seen in the Venn diagram, 11,899 genes were commonly expressed in all the groups (Figure 3A). There were 369 genes differentially expressed in the THP1 co-cultured with control ECs, 177 in the THP1 co-cultured with senECs, and 245 in the THP1 co-cultured with EGCG-treated senECs. To investigate changes at the single gene level involved in the inflammation pathways, we first analyzed all genes associated with various inflammatory processes (Supplementary Figure 3). A curated list of key genes (such as *CXCL8*, *IL-1β*, *C-C motif chemokine ligand [CCL5]*, *TLR4*, *NF-κB1*, *IL-6*) was then compiled to generate a comprehensive heatmap, highlighting expression patterns under different experimental conditions (Figure 3B). In THP1 cells co-cultured with senECs, the expression of most inflammation-associated genes was elevated compared to THP1 mono-culture, THP1 co-culture with control ECs or co-culture with EGCG treated senECs. Notably, THP1 cells co-cultured with EGCG-treated senECs showed a reversal of inflammation-associated gene expression, displaying a profile similar to that of THP1 cells co-cultured with control ECs (Figure 3B).

Gene set enrichment analysis (GSEA) comparing transcript abundance in THP1 cells co-cultured with senECs compared to those expressed in THP1 cells co-cultured with control ECs using the Hallmark database showed that 32 Hallmark gene sets were significantly enriched (*p*adj < 0.05) and 5 gene sets were significantly depleted in THP1 cells co-cultured with senECs (Supplementary Figure 4A). Furthermore, a comparison between THP1 cells co-cultured with EGCG-treated senECs and THP1 cells co-cultured with untreated senECs showed that no gene sets were significantly enriched and 5 gene sets were significantly depleted in THP1 cells co-cultured with EGCG-treated senECs (Supplementary Figure 4B). Highly enriched pathways in THP1 cells co-cultured with senECs were tumor necrosis factor (TNF)-α signalling via NF-κB, inflammatory response, oxidative phosphorylation, and reactive oxygen species pathway (Figure 3C,E, left panels). In contrast, THP1 cells co-cultured with EGCG-treated senECs showed depletion in these signaling pathways (Figure 3D,F, right panels). Overall, these results indicate that senECs enhance inflammatory response and oxidative phosphorylation pathways of THP1 monocytes, while EGCG treatment partially counteracts this effect.

### 3.4 Characterization of EVs

**Figure 4:**
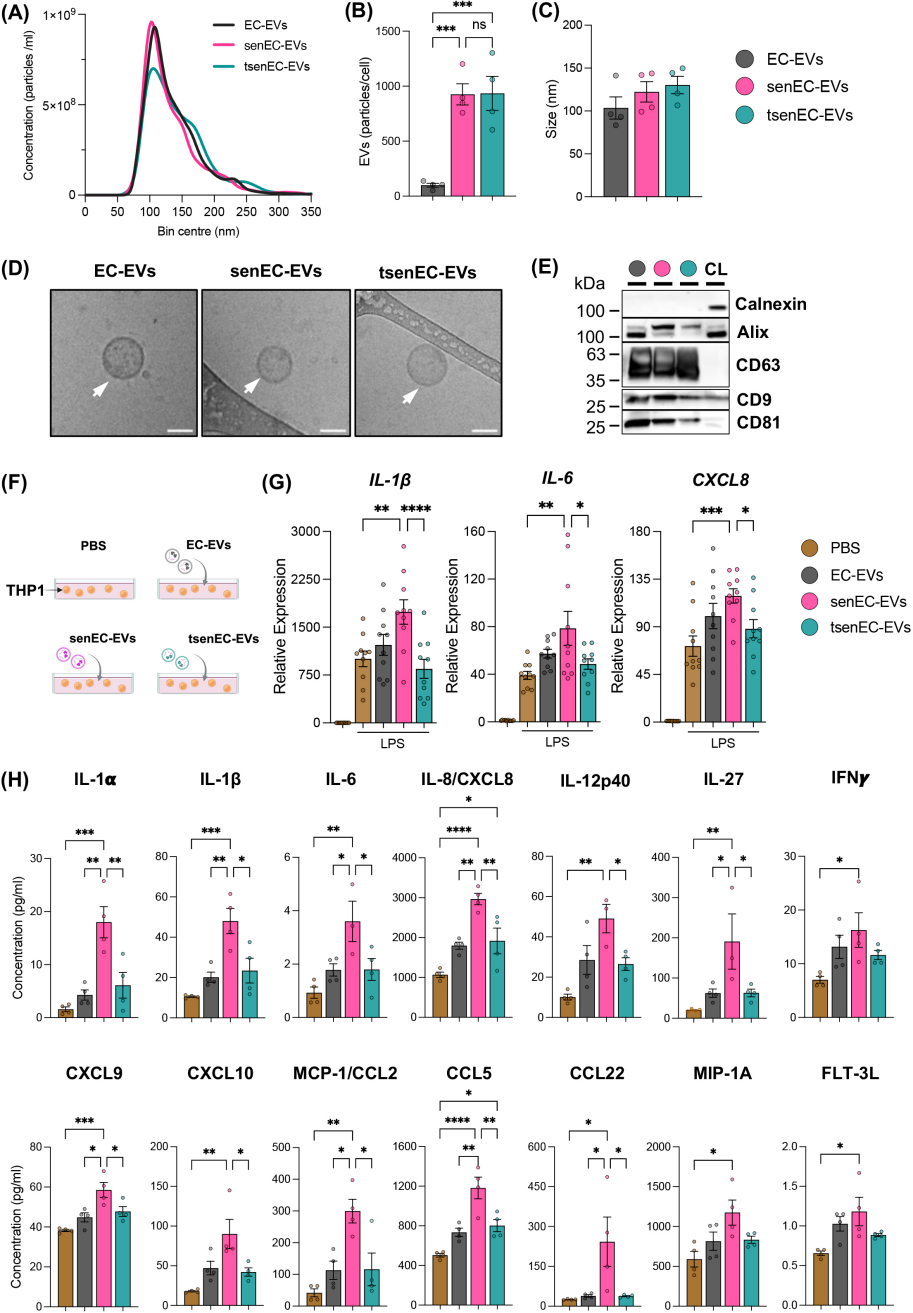
Proinflammatory response of recipient monocytes is differentially modulated by senescent EC-EVs depending on EGCG treatment. **(A)** Nanoparticle tracking analysis (NTA) of EVs isolated from media of control ECs (EC-EVs), senECs (senEC-EVs), and EGCG-treated senECs (tsenEC-EVs) by ultracentrifugation. Quantification of **(B)** EV particles per cell and **(C)** the mode particle size. **(D)** Cryo-EM of EVs isolated from control ECs, senECs, and EGCG-treated senECs supernatant. Arrows indicate EV structures. Scale bar = 50 nm. **(E)** Western blot depicting EV markers [positive (CD63, CD9, CD81 and Alix) and negative (Calnexin)] in EV lysates (EC-EVs, senEC-EVs and tsenEC-EVs) and HUVEC cell lysate (CL) control. **(F)** Schematic of direct EV (PBS, EC-EVs, senEC-EVs, tsenEC-EVs) exposure (24 h) to monocytes (THP1). **(G)** The effect of EVs (EC-EVs, senEC-EVs, tsenEC-EVs) on THP1 cells was assessed by measuring the transcriptional expression of proinflammatory genes in THP1 cells after 2 h of LPS stimulation, using qRT-PCR. Pro-inflammatory markers include *IL-1β*, *IL6*, and *CXCL8*. Data are normalized to the *GAPDH* reference gene. Results are reported as the fold change relative to THP1 (PBS-treated and without LPS treatment). **(H)** Concentration of cytokines/chemokines (IL-1α, IL-1β, IL-6, IL-8, IL-12p40, IL-27, IFNγ, CXCL9, CXCL10, MCP-1/CCL2, CCL5, CCL22, MIP-1A and FLT-3l) in culture media from THP1 cells across all treatment conditions was quantified using human cytokine and chemokine array. Data are presented as mean ± SEM. *n* = 4–5 independent experiments. Statistical analysis was performed using one-way ANOVA with Tukey's multiple comparison test. *p < 0.05, **p < 0.01, ***p < 0.001, and ****p < 0.0001.

We isolated EVs from control ECs (EC-EVs), senECs (senEC-EVs), or EGCG-treated senECs (tsenEC-EVs) via ultracentrifugation from conditioned media. To confirm that these nanoparticles were EVs, we performed nanoparticle tracking analysis (NTA), cryo-EM and western blot (MISEV2023) (49). Quantitative and morphological characterization of EVs was conducted using NTA and cryo-EM (Figure 4A–D). SenECs yielded more EVs (senEC-EVs) than control ECs (EC-EVs) (Figure 4B), with no discernible difference in particle size (Figure 4C). Notably, EGCG treatment did not alter the production or size of particles compared to untreated senECs (Figure 4B,C). As per MISEV guidelines, western blot confirmed the presence of standard EV markers including Alix, CD9, CD63, and CD81, and the absence of calnexin EVs (Figure 4E, Supplementary Figure 5).

### 3.5 Senescent EC-EVs induce a pro-inflammatory phenotype in monocytes that can be mitigated by EGCG

To elucidate the role of EVs in the interaction between ECs and THP1 monocytes, THP1 cells were exposed to different EVs (EC-EVs, senEC-EVs, or tsenEC-EVs) or PBS control for 24 h (Figure 4F). THP1 cells were stimulated with LPS for 2 h after EV treatment. Monocytes exposed to EC-EVs showed no changes in *IL-1β*, *IL6*, or *CXCL8* mRNA expression levels compared to the PBS-treated control cells while exposure to senEC-EVs resulted in significantly increased *IL-1β*, *IL6*, and *CXCL8* mRNA expression (Figure 4G). Interestingly, exposure to tsenEC-EVs significantly reversed the increased *IL-1β*, *IL6*, and *CXCL8* mRNA expression (by 1.7-fold, 2-fold, and 1.7-fold respectively), reverting back to the levels observed in non-senescent EC-EVs-exposed monocytes and PBS-treated controls (Figure 4G).

To further determine the role of EVs on the pro-inflammatory response of THP1 cells, we measured pro-inflammatory cytokine and chemokine production (Figure 4H). Exposure to senEC-EVs increased pro-inflammatory cytokine and chemokine (e.g., IL-1α, IL-1β, IL-6, IL-8, IL-12p40, IL-27, IFNγ, CXCL9, CXCL10, MCP-1/CCL2, CCL5, CCL22, MIP-1A and FLT-3l) protein secretion from monocytes compared to PBS-treated control cells. In contrast, exposure to tsenEC-EVs mitigated these effects, with secretion of several cytokines and (e.g., IL-1α, IL-1β, IL-6, IL-8, IL-12p40, IL-27, CXCL9, CXCL10, MCP-1/CCL2, CCL5, and CCL22) reversed back to levels secreted by PBS-treated monocytes or monocytes exposed to non-senescent EC-EVs. Notably, EGCG reversed IL-1β, IL-6, and IL-8 by 2-, 2-, and 1.5-fold respectively. These findings suggest that EVs derived from senECs enhance LPS-induced activation in THP1 cells, indicating an increased pro-inflammatory effect. In contrast, EVs from EGCG-treated senECs mitigate this activation, maintaining monocyte activation at normal levels. Together, these data suggest that EVs from senescent ECs can transfer cargo to recipient monocytes capable of altering cellular profiles and promoting a pro-inflammatory response. Treatment of senescent ECs with EGCG appears to counteract this pro-inflammatory effect, limiting monocyte activation and response to LPS stimulation.

## 4 Discussion

This study demonstrates that senescent ECs communicate with monocytes to increase pro-inflammatory activity, and that EGCG supplementation of ECs can reverse this proinflammatory communication. An increase in monocyte inflammation has implications for age-related diseases, which underlie atherosclerosis and other cardiovascular pathologies. Our results indicate that EGCG mitigates senescence-associated phenotypes in ECs and increases the production of anti-inflammatory cytokine/chemokines from senescent ECs. Furthermore, monocytes co-cultured with EGCG-treated senescent ECs exhibited reduced pro-inflammatory responses compared to those with untreated senescent ECs, indicating that EGCG treatment restricts monocyte activation induced by the secretome of senescent ECs that enhance inflammation. Notably, senescent ECs elevate monocyte gene expression in various pathways related to inflammation and oxidative phosphorylation, but EGCG treatment can halt this gene expression; thus, these data demonstrate that EGCG treatment can reverse EC senescent programming and restore homeostatic EC-monocyte communication. Moreover, senescent ECs produced more EVs than control ECs. We showed that EVs from senescent ECs enhance LPS-induced pro-inflammatory activation in monocytes, while EVs from EGCG-treated senescent ECs mitigate this activation, maintaining monocyte activation at normal levels. Overall, these data suggest that EGCG appears to have a senomorphic effect on senescent ECs, impacting their secretome—including EVs—leading to reversal of the increased monocyte activation and inflammation induced in response to co-culture with senescent ECs. Reversal of endothelial senescence and restoration of homeostatic EC-monocyte crosstalk by EGCG should be explored further in the context of age-related diseases.

Etoposide was used for this study due to its well-established role in inducing senescence by targeting DNA double-strand breaks (50, 51), making it an ideal tool for investigating senescence-related processes. Our study showed Etoposide-induced a senescent phenotype in ECs. EGCG, a compound that influences numerous molecular pathways, has potential benefits in treating various diseases such as cancer, neurological, cardiovascular, respiratory, and metabolic disorders (52). SASP factors such as pro-inflammatory and immune-modulatory cytokines, chemokines, proteases, and growth factors change during cellular senescence (48). Numerous pro-inflammatory cytokines and growth factors, including GM-CSF, IL-1, and IL-6 and anti-inflammatory cytokines such as IL-4 and IL-13 are produced by senescent cells (48, 53). Here, we demonstrated that EGCG treatment in senescent ECs reduces senescent markers, including SASP. We also evaluated the efficacy of two other senolytic/senomorphic agents, quercetin and resveratrol, in mitigating EC senescence. However, EGCG exhibited superior anti-senescent efficacy compared to alternative agents within our experimental model (Supplementary Figure 2).

Our data indicate that senescent ECs increase pro-inflammatory factors and decrease anti-inflammatory factors, a shift reversed by EGCG treatment, suggesting its immunomodulatory impact in endothelial senescence. EGCG has senolytic and senomorphic effects through various mechanisms (38, 39). EGCG inhibits the premature senescence of preadipocytes by suppressing the PI3K/Akt/mTOR pathway and promotes senescent cell death through the modulation of the Bax/Bcl-2 pathway (38). Additionally, EGCG exhibits senomorphic effects by reducing SASP via the activation of SIRT3 in preadipocytes (39). Our findings demonstrate that EGCG treatment significantly reduced all indices of senescence compared to senescent ECs (Figure 1A–D). Compared to non-senescent controls, EGCG fully reversed most but not all markers back to baseline, implying senomorphic potential and in keeping with other emerging senomorphics that dampen the spectrum of SASP (54). Consistent with earlier findings (8), our data showed a decrease in pro-inflammatory responses in monocytes co-cultured with untreated ECs. Specifically, EVs from untreated ECs inhibited the activation of proinflammatory markers (such as IL-12p40, IL-23p19, TNF-α, and IL-1β, while promoting the expression of markers associated with anti-inflammatory response (such as IL-10, MRC1, and TGF-β). We demonstrate for the first time that senescent ECs communicate with monocytes, resulting in increased pro-inflammatory responses in co-cultured monocytes, which can be mitigated by treating senescent ECs with EGCG. Other studies have shown the biological effects of EGCG in targeting molecular pathways governing inflammation and oxidative stress (52). Our results showed a similar effect: EGCG treatment of senescent ECs alters communication with monocytes to suppress the activation of inflammatory pathways and oxidative phosphorylation compared to non-treated senescent ECs. Others have demonstrated that EGCG downregulates several components of the TNF-α-induced NF-κB signalling pathway, thereby reducing the inflammatory response in ECs (55). Additionally, EGCG can protect vascular ECs from oxidative stress-induced damage by modulating the autophagy-dependent PI3K-AKT-mTOR pathway (44). Our findings, together with previous studies, suggest that EGCG can influence these pathways in senescent ECs, altering their communication with monocytes and resulting in decreased activation of inflammatory pathways and oxidative phosphorylation. Previous studies have demonstrated that ECs-EVs modulate the monocyte/macrophage phenotype (8, 28–31). Here, we have shown for the first time how senescent ECs communicate differently with monocytes through EC-EVs. Our results show that exposure to senescent EC-EVs increases the pro-inflammatory response in monocytes, while EVs from EGCG-treated senescent ECs restore this response to baseline levels. ECs have been shown to release different EV cargo (e.g., miRNA, protein) when they undergo senescence (56–58), and that these can propagate senescence to recipient ECs (56, 59). Other work has demonstrated that EVs derived from senescent vascular smooth muscle cells are carriers of SASP components and can influence monocyte inflammatory responses (60). Our data indicate that although EGCG treatment does not alter the size or number of EVs produced by senescent ECs, it significantly reduces monocyte activation responses. Further studies are needed to confirm whether senEC-EVs specifically carry SASP-associated cytokines and chemokines and to explore the modulatory effects of EGCG on EV composition and function.

A limitation of this study is the use of a drug (Etoposide)-induced model, which offers rapid induction of senescence. While this approach effectively activates senescent programming in ECs, it may not fully represent the complex and multifactorial processes involved in senescence associated with natural aging. As a DNA-damaging agent, Etoposide may also activate non-senescent stress responses in ECs. Alternative approaches, such as replicative senescence models or oxidative stress-induced senescence models, could provide a more comprehensive understanding of these processes. There are other markers of senescence that could be tested in future experiments. We utilized HUVECs and THP1 cells in this study, acknowledging that while HUVECs may not capture the diverse characteristics of ECs from various vascular beds, and THP1 cells may not fully replicate the behavior and heterogeneity of primary human monocytes *in vivo*, both cell lines provide reliable *in vitro* data. Their established roles in cell biology allow for meaningful comparisons with existing research. A fundamental limitation of working with EVs is the difficulty in ensuring the purity and consistency of isolated EV populations, as contaminants like proteins or other vesicle types can interfere with experimental results. Additionally, the heterogeneity of EVs in size, cargo, and function can make it challenging to draw definitive conclusions about their specific roles in cell signalling or disease processes. Finally, future work could explore the EV cargo released from senescent ECs and specifically determine how this cargo is altered by treatments that reverse senescent programming.

CVDs and atherosclerosis are global health issues significantly impacted by aging (61). One of the primary contributors to these conditions is monocyte inflammation and oxidative phosphorylation (62, 63). This study suggests a potential contribution of senescent ECs to this process. Here, we present *in vitro* data indicating that EGCG treatment mitigates the senescent EC secretome contributing to underlying processes that can be associated with CVD and atherosclerosis, positioning it as a potential therapeutic agent. Interestingly, EGCG does have direct effects on ECs, where it has been shown to scavenge reactive oxygen species and exert antiangiogenic, and antithrombotic effects in a murine tumor model (64). Investigating how EGCG-treated senescent ECs affects other monocyte functions, such as adhesion and migration, is a crucial next step in evaluating its potential as a therapeutic agent for age-related diseases like atherosclerosis. As previously shown in a study from our lab, identifying alterations in EV cargo can facilitate new therapeutic discoveries (28); thus, further investigation is necessary to determine how EGCG may modulate EV cargo, including proteins and miRNAs, in the context of developing age-related disease therapies.

With this study, we begin to understand how aging affects intercellular communication within the hemothelium and the potential role of senescent EC-EVs in age-related diseases. This study determined the inflammation mediating impact of EGCG on senescent EC-monocyte communication. We anticipate that EGCG treatment can reduce senescent EC-EV-induced monocyte dysfunction, which can be beneficial in the development of new strategies to interrupt or mitigate senescence. Carefully designed *in vivo* studies will ultimately be required to test the therapeutic utility of EGCG for age-related vascular diseases.

## Data Availability

The data presented in the study are deposited in the Gene Expression Omnibus, reference number GSE286438.
