# Supplementary material for: Epigallocatechin gallate (EGCG) modulates senescent endothelial cell-monocyte communication in age-related vascular inflammation
Source: Front Cardiovasc Med. 2025 Jan 21;11:1506360. doi: 10.3389/fcvm.2024.1506360 (PMC11790594; doi:10.3389/fcvm.2024.1506360)
Supplement: Supplementary file 1 [file Datasheet1.docx]

Supplementary Material

# Supplementary Figures and Tables

**Supplementary Table 1. Primers list for the qRT-PCR.**

**Supplementary Figure 1. Effects of Etoposide on HUVECs to induce senescence.** HUVECs were treated with Etoposide (10µM) for 6h, 12h and 24h to induce senescence. **(A)** Representative cellular morphology and SA-β-gal staining of HUVECs under various conditions - (i) No treatment (control), (ii) Treated with Etoposide (10µM) for 6h, 12h, and 24h (senescence). **(B)** qRT-PCR analysis of senescence-related genes *CDKN1A*, *CDKN2A*, *CDKN2B*. **(C)** qRT-PCR analysis of senescence-associated secretory phenotype (SASP)-related genes *CXCL8*, *IL6*. Data are given as ± SEM. n=3. Statistical analysis was performed using the unpaired t-test. NT, no treatment; ns, not significance; **p <* 0.05, ***p <* 0.01, ****p <* 0.001 and *****p <* 0.0001.

**Supplementary Figure 2. Effects of senotherapeutics on senescent HUVECs.** HUVECs were treated with Etoposide (10µM) for 24h to induce senescence, followed by treatment with or without Quercetin (5µM), Resveratrol (10µM), or EGCG (100µM) for 24h. **(A)** Representative cellular morphology and SA-β-gal staining of HUVECs under various conditions - (i) No senotherapeutics, Treated with (ii) Quercetin (5µM), (iii) Resveratrol (10µM), or (iv) EGCG (100µM). **(B)** Quantification of the percentage of SA-β-gal positive HUVECs. **(C)** qRT-PCR analysis of senescence-related genes p21, p16, p15. **(D)** qRT-PCR analysis of senescence-associated secretory phenotype (SASP)-related genes *CXCL8*, *IL6*. **(E)** qRT-PCR analysis of adhesion-related markers, including the genes *SELE*, *VCAM1* and *ICAM1*. Data are given as ± SEM. n=3. Two-way ANOVA with a Tukey’s multiple comparison test; **p <* 0.05, ***p <* 0.01, ****p <* 0.001 and *****p <* 0.0001.

**Supplementary Figure 3. Effect of EGCG treatment on the communication between senescent HUVECs and monocyte inflammatory gene profile.** The impact of co-culturing THP1 cells with HUVECs for 24h was further assessed using RNA sequencing of THP1 cells. **(A)** Heatmap showing the expression profiles of pro-inflammatory genes. Gene expression was assessed across different experimental conditions to evaluate changes in inflammatory responses. Expression values are represented as colours and range from Yellow (high expression), green (moderate), to dark blue (lowest expression). Enrichment plots of inflammation-related gene sets comparing.

**Supplementary Figure 4. Gene set enrichment analysis (GSEA) results of comparison between THP1 co-cultured with different HUVECs.** RNA-Seq was performed on samples collected after 24h co-culturing THP1 cells with different HUVECs (control, senescent and EGCG treated senescent). Results of GSEA Hallmark analysis showing enriched gene sets **(A)** in THP1 cells co-cultured with senescent HUVECs vs. control HUVECs **(B)** THP1 cells co-cultured with EGCG-treated senescent HUVECs vs. senescent HUVECs. Bars in blue indicate significant enrichment at adjusted P value < 0.05, bars in red represent gene sets with adjusted P value < 0.05.

**Supplementary Figure 5. Western blot analysis of EC-EV markers. (A)** Schematic of the Western blot gel used to analyze EV markers, outlining the process for cutting the gel and probing with different antibodies for analysis. **(B)** Images of western blot analysis of different markers of EVs (positive: Alix, CD63, CD81, CD9 and negative: Calnexin)
